# Cytomegalovirus Retinitis in a Patient Taking Upadacitinib: A Case Report

**DOI:** 10.7759/cureus.48337

**Published:** 2023-11-05

**Authors:** Hiromasa Hirai, Yasuhiro Akai, Nahoko Ogata, Tetsuo Ueda

**Affiliations:** 1 Ophthalmology, Nara Medical University, Kashihara, JPN; 2 Community Medicine, Rheumatology, Nara Medical University, Kashihara, JPN

**Keywords:** jak inhibitor, rheumatoid arthritis, upadacitinib, cytomegalovirus (cmv), cytomegalovirus retinitis

## Abstract

Upadacitinib is a relatively new drug used to treat autoimmune diseases. However, patients treated with upadacitinib may develop infections. We report a case of cytomegalovirus (CMV) retinitis that developed during upadacitinib administration. A 79-year-old woman presented with progressively decreasing vision in both eyes. Her decimal best-corrected visual acuity (BCVA) was 0.2 in the right and 0.01 in the left eye. The patient was taking upadacitinib for one year. Fundus examination revealed vitreous opacities and extensive white retinal lesions with hemorrhage in both eyes. CMV was detected in the anterior aqueous humor, vitreous humor, and blood samples. We diagnosed her with panuveitis and CMV retinitis, performed a vitrectomy in both eyes, and administered intravenous ganciclovir and steroids. After treatment, her BCVA improved to 0.6 in the right and 0.1 in the left eye. Ophthalmologists and physicians should be aware of CMV infections in patients being treated with upadacitinib.

## Introduction

Upadacitinib is used for the treatment of autoimmune diseases such as rheumatoid arthritis [1]. Patients taking this drug sometimes develop side effects, including infection [1,2]; however, ocular infections are rare. Human cytomegalovirus (CMV) also known as human herpesvirus 5 is a common infection [3]. CMV usually infects during childhood and remains asymptomatic throughout life in healthy people, and more than 60% of adults have CMV-specific IgG antibodies [3]. CMV activates in immunosuppressed patients, resulting in severe organ damage. It causes pneumonia, gastrointestinal disease, hepatitis, and retinitis [3,4]. CMV retinitis, characterized by retinal white lesions with hemorrhage, may result in permanent vision loss [4]. Early diagnosis of CMV retinitis is important for patients’ quality of life. We describe the case of a patient with CMV retinitis treated with upadacitinib for rheumatoid arthritis.

## Case presentation

A 79-year-old woman presented with progressively decreasing vision in both eyes. She noticed vision loss and visited her local eye clinic four months ago. The patient was diagnosed with idiopathic uveitis and treated with betamethasone eye drops and subtenon injections of triamcinolone acetonide. However, her vision worsened, and she was referred to our hospital. Her medical history included rheumatoid arthritis, Sjögren syndrome, interstitial pneumonia, asthma, and type 2 diabetes mellitus on insulin. She was diagnosed with rheumatoid arthritis 22 years ago, treated with a combination of oral prednisone and methotrexate, and received intravenous infliximab every eight weeks. She was diagnosed with mild interstitial pneumonia 11 years ago and underwent follow-up chest computed tomography scans and blood examinations. Moreover, she was diagnosed with Sjögren syndrome eight years ago. Her dry eye and dry mouth symptoms were mild. She was infected with severe acute respiratory syndrome coronavirus 2 (SARS-CoV-2) two years ago and was also diagnosed with post-coronavirus interstitial pneumonia. The use of methotrexate and infliximab was discontinued to prevent exacerbation of interstitial pneumonia. However, she complained of swelling and pain at the metacarpophalangeal joints and proximal interphalangeal joints in both hands. Therefore, oral upadacitinib (15 mg/day) was started one year ago. The swelling and pain in her fingers improved, and she had no signs of any new infection or respiratory symptoms. Moreover, she underwent cataract surgery in both eyes at the initial eye clinic one year ago. Her postoperative best-corrected visual acuity (BCVA) was 1.0 in both eyes.

**Figure 1:**
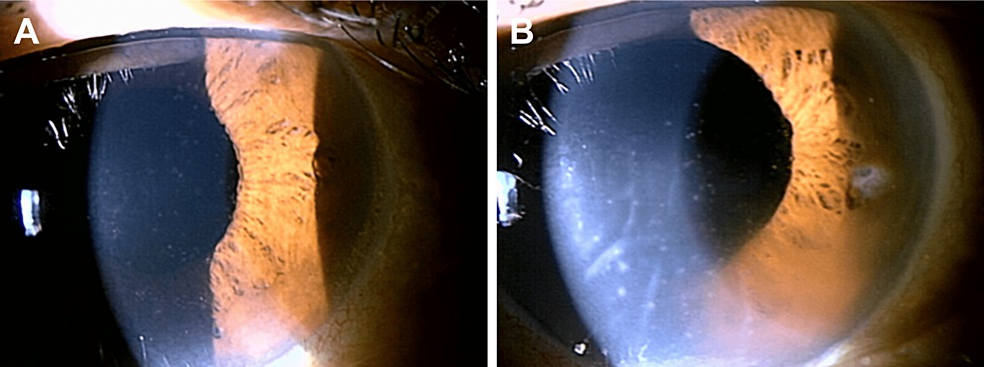
Photographs of the anterior segment of the eyes at the initial visit. A. Right eye. Fine keratic precipitates (KPs) were observed. The cornea remained transparent. B. Left eye. Corneal edema and Descemet's folds were present. Large pigmented KPs were observed.

At our initial examination, her BCVA was 0.2 in the right and 0.01 in the left eye. The intraocular pressure was 11 mmHg in the right and 12 mmHg in the left eye. Slit-lamp examination revealed corneal edema, Descemet folds, and several pigmented keratic precipitates (KPs) in the left eye (Figure 1).

**Figure 2:**
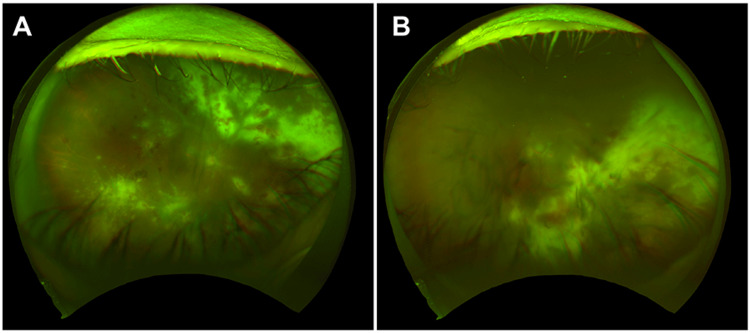
Fundus photographs at the initial visit. A. Right eye. White retinal lesions with hemorrhage were observed on the superior nasal and inferior temporal areas. The photograph was slightly obscured because of vitreous opacity. B. Left eye. White retinal lesions extending from the macula area to the temporal side were observed. The photograph was slightly obscured due to vitreous opacity.

Therefore, the left corneal translucency was poor. The cell gradings of the anterior chamber were 3+ in both eyes [5]. Fundus examination revealed vitreous opacities and extensive white retinal lesions with hemorrhage in both eyes (Figure 2).

**Figure 3:**
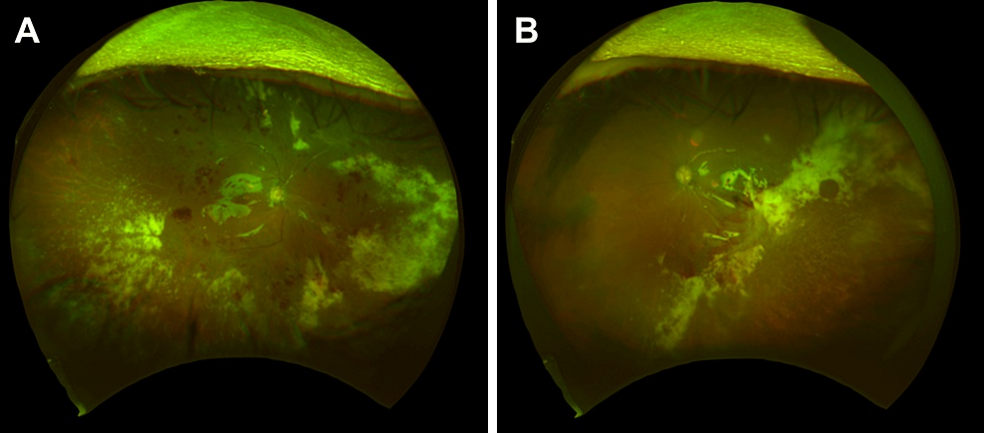
Fundus photographs at the discharge. A. Right eye. After silicone oil tamponade. The white retinal lesions were improved compared to the initial examination. B. Left eye. After silicone oil tamponade. The white retinal lesions were improved compared to the initial examination.

We collected anterior aqueous humor for polymerase chain reaction (PCR) examination. The CMV antigenemia assay method (C7-HRP) at the initial blood examination was positive (223 positive cells/50,000 leukocytes) and confirmed active CMV infection. Her body temperature was within normal range. The patient had no systemic symptoms other than finger stiffness. The patient was diagnosed with panuveitis and CMV retinitis. We hospitalized her, discontinued upadacitinib therapy, and started treatment with intravitreal ganciclovir (280 mg/day) and prednisone (60 mg/day). We measured her blood glucose four times a day and adjusted the units of insulin. Moxifloxacin, betamethasone, nepafenac, and ganciclovir eye drops were administered. We performed a vitrectomy combined with posterior hyaloid removal and silicone oil tamponade in the right eye on day two. We collected vitreous humor during the surgery. We also administered intravitreal injections of foscarnet in the right (day one) and left eye (days one and eight). Both PCR examinations of anterior aqueous and vitreous humors confirmed CMV. Inflammation gradually decreased, and the retinal lesion showed a tendency to regress. As C7-HRP levels improved (three positive cells/50,000 leukocytes) on day 14, we switched to oral valganciclovir (900 mg/day) on day 16. We gradually decreased the prednisone dose and changed it to oral administration. She was infected with SARS-CoV-2 on day 16 and had a slight fever and mild cough. However, her oxygen saturation (SpO2) was within normal range and her chest X-ray showed no changes. The patient was isolated until SARS-CoV-2 negative confirmation and was treated with oral molnupiravir for five days. After the left cornea became clear, we performed a vitrectomy combined with posterior hyaloid removal and silicone oil tamponade in the left eye on day 30. We reduced the amount of valganciclovir (450 mg) after two negative CMV confirmations on blood examinations (on days 24 and 30) and prednisone (20 mg/day). The patient was discharged on day 39 with an improved BCVA, 0.6 in the right, and 0.1 in the left eye (Figure 3).

We obtained written informed consent from the patient for the publication of this case report, which does not contain any personal identifying information.

## Discussion

We describe a case of CMV retinitis during oral treatment with upadacitinib. Upadacitinib is a Janus kinase (JAK) inhibitor [1,6]. JAK is a group of intracellular enzymes involved in the signaling of inflammatory cytokines, such as interferons and interleukins [1]. JAK has four isoforms (JAK1, JAK2, JAK3, and tyrosine kinase 2), and upadacitinib is a selective JAK1 inhibitor [6]. Upadacitinib was approved in the United States in 2019 as a new drug to treat moderate-to-severe rheumatoid arthritis, an autoimmune disease characterized by inflammation and bone destruction [7,8]. Besides rheumatoid arthritis, upadacitinib is currently used to treat several autoimmune diseases [8-10]. However, upadacitinib sometimes causes side effects, including neutropenia, hepatic disorder, venous thrombosis, and infections [1,8]. A phase 3 study of upadacitinib reported that 24 weeks of upadacitinib administration (15 mg/day) caused candidiasis (1.3%) and herpes zoster (1.3%) [2]. Few reports are available regarding CMV infections in the same herpes genus. A clinical trial documented a patient who experienced a CMV infection, and whether this individual developed retinitis remains uncertain [8]. Although a few reports of CMV retinitis were noted in patients taking tofacitinib (a selective JAK1, JAK2, and JAK3 inhibitor) [11,12], no similar case reports of upadacitinib were found in the PubMed database. CMV retinitis is an opportunistic infection often observed in patients with acquired immunodeficiency syndrome [4]. CMV retinitis is also observed in patients undergoing hematopoietic stem cell transplantation and blood disorders [4,13]. A recent study reported that CMV antigenemia was found in patients with autoimmune diseases, including systemic lupus erythematosus, Sjögren syndrome, and rheumatoid arthritis [14]. The long immunosuppressive conditions in patients with the treatment for those autoimmune diseases may activate potential CMV infection, resulting in organ damage. CMV retinitis can also occur due to topical administration of steroids [15,16]. Although the patient also received topical steroids, her initial blood examination revealed a systemic CMV active infection, implying that long-term oral upadacitinib administration might have contributed to the systemic infection. Anti-CMV drugs (intravenous ganciclovir or oral valganciclovir), intravitreal injections of anti-CMV drugs, and vitrectomies are usually performed to treat CMV retinitis [4]. In some cases, steroids can also be administered [17]. In this case report, the patient had several autoimmune systemic diseases (such as severe rheumatoid arthritis and interstitial pneumonia). She also presented with panuveitis and severe inflammation in both eyes. Therefore, the patient received a combination of systemic steroids and anti-CMV drugs for treatment, resulting in the resolution of inflammation and improved visual acuity.

## Conclusions

We report the development of CMV retinitis in a patient on upadacitinib. Ophthalmologists and physicians should exercise caution regarding CMV retinitis in patients being treated with upadacitinib. Their close collaboration is crucial for effective patient monitoring and management.
